# Oral foci of infection in pediatric population during COVID-19 omicron pandemic era 

**DOI:** 10.3205/dgkh000456

**Published:** 2024-01-30

**Authors:** Manikandan Gunasekaran, Karthik Shunmugavelu, Kavitha Ponnusamy, Aditya Shinde, Selvam Azhagarsamy, Shobana Murali

**Affiliations:** 1 Dept of Dentistry, Shri Sathya Sai Medical College and Research Institute, Ammapettai village, Kancheepuram district, Tamilnadu, India; 2 PSP Medical College Hospital and Research Institute Tambaram Kanchipuram main road Oragadam Panruti Kanchipuram district, Tamilnadu, India; 3 Government Omandurar Medical College, Chennai, Tamilnadu, India; 4 MGM Dental College, Navi Mumbai, India; 5 Department of Dental surgery, Government Theni medical college, Theni, Tamilnadu, India; 6 Ragas Dental College and Hospital, Chennai, Tamilnadu, India

**Keywords:** dental caries, paediatric patients, prevalence, SARS-CoV-2, Covid-19, omicron, therapy

## Abstract

Caries is a multifactorial disease that involves a majority of the pediatric population. If not diagnosed and treated, it can lead to severe consequences affecting the permanent dentition. The objective of this study is to assess the prevalence of oral foci of infection in a multispeciality hospital during pandemic in Chennai, South India. Majority of the patients examined had caries.

## Abstract (de)

Karies ist eine multifaktorielle Erkrankung, von der ein Großteil der pädiatrischen Bevölkerung betroffen ist. Wenn sie nicht diagnostiziert und behandelt wird, kann sie schwerwiegende Folgen für das bleibende Gebiss haben. Ziel dieser Studie ist es, die Prävalenz oraler Infektionsherde in einem multidisziplinären Krankenhaus während der Pandemie in Chennai, Südindien, zu untersuchen. Die Mehrheit der untersuchten Patienten hatte Zahnkaries.

## Introduction

Caries is a multifactorial, biofilm-mediated, diet modulated, non-communicable and dynamic disease that results in net mineral loss of dental hard tissues [1]. “Dental caries” originates from the Latin word “caries” which means for decay. It is a multifactorial disease that is primarily associated with Streptococcus mutans that demineralize the tooth structure [2]. The most commonly accepted theory for the disease aetiology is the ‘chemo-parasitic theory’ proposed by W. D. Miller in 1891 [3]. He suggested that the combined effects of acids and the oral microorganisms results in tooth decalcification [4], [5].

Approximately 2.4 billion of the world population have permanent teeth affected by dental caries. More than 530 million of pediatric population lose their primary teeth due to dental caries [5],[6], [7]. 

In children, the prevalence of dental caries is affected by several factors including socio economic status, inappropriate dietary factors and altered immune response. This article aims to assess prevalence of dental caries at a multispeciality hospital during pandemic in Chennai, South India. The treatment involving dental caries management were also assessed.

## Materials and methods

This study was performed from 2020–2021. A total of 257 paediatric patients were assessed. Demographics, chief complaint, history of presenting illness, past medical history, extra oral and intra oral examination were assessed. Intraoral examination details involved assessment of hard tissue, soft tissue and radiographic examination. Dental caries was diagnosed based on clinical examination and radiographs. DMFT index was used to assess the caries involvement. Caries disease indicators such as visible cavitations, active white-spot lesions, interproximal radiographic lesions penetrating to the dentin, and a history of any cavitations were also assessed. An explorer which catches or resists removal when moderate pressure is applied was used as a clinical indicator of dental caries. The dental caries associated management was also assessed. The results were collected and analysed with descriptive statistics using Statistical Package for Social Sciences (SPSS version 2).

## Results

### Demographics

The gender distribution was 49% male and 51% female.

### Prevalence of dental caries

The prevalence of dental caries was 82.1%. Out of 257 patients examined, 211 patients presented with dental caries.

### Site of the lesion

Most of the dental caries diagnosed where present in the occlusal surface (65%), followed by incisal (24%) and the distal surface (11%).

### Management of dental caries

The majority of the lesions were treated by restorations (76%) followed by extractions (20%), prosthetic replacement (3%) and orthodontic treatment (1%).

## Discussion

Out of the 257 paediatric patients examined, 211 had presence of dental caries. There was no significant difference in the gender distribution. The diagnosis of carious lesions has been primarily a visual process, based principally on clinical inspection and review of radiographs. Tactile information obtained through use of the dental explorer or “probe” has been used in the diagnostic process. A majority of the diagnosed dental caries was present on the occlusal surfaces followed by the incisal and proximal surfaces. This can be attributed to improper brushing technique, lack of poor oral hygiene and inappropriate diet such as sucking on toffees and candy bars. The majority of the lesions were treated by restorations. However, extractions were also carried out followed by prosthetic replacement. Orthodontic treatment was done in only one of the paediatric patients. Convenience sampling is a limitation to this study. The data for the purpose of the study was collected from respondents in Chennai only. The other limitation includes lack of proper diagnostic scale.

## Conclusion

Dental caries one of the most common dental problems in toddlers and children. If not diagnosed and treated, it can lead to severe consequences affecting the permanent dentition too. Fluoridation and proper oral hygiene technique must be taught.

## Notes

### Author’s ORCID

- Karthik Shunmugavelu: 0000-0001-7562-8802
